# Severity of Trachomatous Scarring among Adults in Trachoma-Endemic Amhara Region of Ethiopia

**DOI:** 10.4269/ajtmh.23-0894

**Published:** 2024-06-25

**Authors:** Jaymie A. Bromfield, Ugochi T. Aguwa, Eshetu Sata, Kimberly A. Jensen, Fetene Mihretu, E. Kelly Callahan, Sheila K. West, Meraf A. Wolle, Scott D. Nash

**Affiliations:** ^1^ Trachoma Control Program, The Carter Center, Atlanta, Georgia;; ^2^ Dana Center for Preventive Ophthalmology, Wilmer Eye Institute, Johns Hopkins Hospital, Baltimore, Maryland;; ^3^ Trachoma Control Program, The Carter Center, Addis Ababa, Ethiopia

## Abstract

Trachomatous scarring has been shown to progress regardless of active ocular *Chlamydia trachomatis* infection, indicating that scarring drivers may be unrelated to ongoing transmission. Although scarring prevalence is commonly associated with older age and female sex, less is known about other potential contributors to its development. This study identified and assessed risk factors associated with scarring magnitude in a trachoma-endemic setting, utilizing a five-point photographic scale (S0–S4). During 2017 trachoma surveys of Amhara, Ethiopia, photographers captured left and right conjunctival images of adults (ages 15 years and older) from 10 districts. Subsequently, two graders independently assessed photographs for scarring, with discrepancies adjudicated by an expert grader. Scarring scores for 729 individuals were aggregated from the eye level to the participant level, excluding 17 participants because of poor photograph quality. Among those with scarring, most cases (20.4%) were severe (S4, comprising more than 90% of the tarsal conjunctiva) compared with the prevalence of moderate S3-A/B (11.2%), S2 (8.3%), and mild S1 (19.2%). The youngest group (ages 15–19 years) exhibited all scarring stages. Older participants (60 years and older) experienced a greater burden of severe scarring (S4 prevalence: 32.6%) than their younger (15–19 years) counterparts (6.2%). Multivariate ordinal logistic regression models indicated female sex, increasing age, and district-level trachomatous follicular–inflammation prevalence were significant predictors of scarring severity. Trachomatous scarring and its progression to trichiasis, may prove a barrier to meeting WHO timelines for trachoma elimination and will necessitate ongoing surveillance and interventions after elimination thresholds have been met.

## INTRODUCTION

Trachoma is caused by repeated ocular infection with the bacterium *Chlamydia trachomatis*.^1^ Active trachoma, or the presence of clinical signs of trachomatous inflammation*–*follicular (TF) or trachomatous inflammation*–*intense, is commonly observed in childhood because of the transmission of *C. trachomatis* occurring frequently between children.^1^ Ocular *C. trachomatis* transmission is thought to be highest in environments with low water and sanitation availability, and accordingly, the WHO recommended interventions specifically targeting facial cleanliness and environmental improvement alongside mass drug administration (MDA) with antibiotics.^1^ Trachomatous scarring of the conjunctiva is a result of a T helper 2 cell*–*mediated immune response initially triggered by repeated *C. trachomatis* infections and is generally noted to occur in adulthood, although studies within communities of high endemicity have observed scarring in children.^2^^–^^6^ The severity and baseline presence of scarring are strongly associated with the risk of trachomatous trichiasis (TT) development.^7^^,^^8^ If left untreated, TT can cause corneal opacity (CO) and blindness.

In a region-wide population-based study of Amhara, Ethiopia, trachomatous scarring, defined as easily visible scars of the conjunctiva, was shown to increase with age. Prevalence among individuals ages 61*–*65 years reached 24.2%.^5^ Several prevalence studies have also demonstrated that women are more likely to develop signs of TT and trachoma-related blindness than their male counterparts.^9^^–^^12^ In a meta-analysis of more than 12 countries, the odds of TT among women were 1.82 times that among men.^13^ This differential could be due to more scarring at all levels in women or more women with more severe scarring; however, studies of scarring severity by sex are rare.

Although repeated *C. trachomatis* infection is required for the development of conjunctival scarring, once acquired, progression of scarring may in part be independent of ongoing transmission. Progression may be related to inflammatory processes potentially triggered by non-chlamydial bacterial infections or adverse environmental conditions.^3^^,^^4^^,^^14^^–^^19^ Trachoma transmission is currently monitored programmatically through TF prevalence assessments, whereas scarring is not widely assessed. Understanding the correlation between TF and scarring severity may help programs identify areas at risk for high rates of TT. Considerable research efforts have been undertaken to understand the relationships between TF and availability of water, sanitation, and hygiene (WASH).^20^^,^^21^ Less is known about how scarring severity may be related to low-WASH environments common in trachoma-hyperendemic settings such as Amhara.

The aim of this study was to investigate the prevalence and severity of scarring among men and women in a trachoma-hyperendemic setting using survey-collected conjunctival photographs and a validated five-point scarring scale.^2^^,^^17^^,^^18^^,^^22^ Relationships between age, concurrent TF prevalence, and levels of WASH were also elucidated.

## MATERIALS AND METHODS

### Ethical considerations.

The survey protocol was approved by the Emory University Institutional Review Board and the Amhara National Regional State Health Bureau (#079-2006). This survey was conducted in full compliance with the Declaration of Helsinki. Informed oral consent among all participants was collected in 2017 owing to low literacy rates within the population.^23^

### Data collection.

This analysis used conjunctival photographs collected during cross-sectional trachoma impact surveys conducted in January and February of 2017 within the eastern half of Amhara. The sampling and data collection methods were extensively reported previously.^23^^–^^25^ To summarize, a multistage cluster random sampling methodology was used to select villages (clusters) in the first stage of sampling and households in the second stage to estimate trachoma outcomes after 3–8 continuous years of surgery, antibiotics, facial cleanliness, and environmental improvement (SAFE) strategy implementation. Each participant or their consenting guardian provided demographic information on their sex and age in addition to household-level data on WASH indicators, such as presence of a latrine, presence of an improved water source, and distance to and from the water source. In addition, in-field grading of trachoma signs was made by trained and certified graders using the WHO simplified grading scheme. Survey recorders collected data from participants using tablets compatible with an Open Data Kit application.^26^

### Photography.

Photographic methods, including camera specifics and photographer training, were published previously.^23^ Briefly, one trained and certified photographer used a digital SLR camera (Canon EOS 60D, Tokyo, Japan) to photograph all survey participants examined in 10 survey clusters (one in each of 10 survey-eligible districts) (Supplemental Figure 1). The photographer was assigned to work with one of four field graders for each cluster, depending on survey logistics for that given day. The photographer took at least two photographs per eye. Additional photographs were taken at the discretion of the photographer if either was deemed unsuitable, such as being out of focus.

### Photographic grading.

Photographs were graded for the presence of scarring and severity based on a five-point scale developed by Wolle et al.,^2^ which has been shown to be reliable and valid.^22^^,^^27^ The scale ranges from S0 to S4 with subcategories of level S3 (Table 1).^2^^,^^27^ Prior to grading, two graders attended a 3-day training including lectures, analysis of conjunctival photographs, and practice examinations, culminating with a final certification examination where trainees’ independent grades on a series of 60 images were compared with those of two professionals (S. K. West and M. A. Wolle) with extensive experience in trachoma grading. Both graders passed the final examination and were certified with a κ score >0.7.

**Table 1:** Definitions of conjunctival scarring clinical signs

Stage	Description
S1	≥1 Lines of scarring ≥3 mm in length and some stellate scars, but total area involving <1/8th of the upper tarsal conjunctiva
S2	Multiple lines or patches of scarring involving ≥1/8th, but <1/3rd, of the upper tarsal conjunctiva
S3	Scarring of ≥1/3rd of the upper lid with clear conjunctiva between, but involving <90% of the upper eyelid
S3–A	Scarring of ≥1/3rd of the upper lid with clear conjunctiva between, but total scarring involving <50% of the upper eyelid
S3–B	Total scarring involving between 50% and 90% of the upper eyelid
S4	Scarring involving >90% of the conjunctiva

From October 2022 to January 2023, two independent graders assessed two photographs of each conjunctiva concurrently, in random order, of adult participants (15 years and older) at ×5 magnification. Other environmental conditions for grading included dim room lighting, maximum screen brightness, and full resolution of the photograph encompassing the screen. Each grader was masked to all collected demographic information, the eye-level identification number of each photo set, and the assessment of the other grader.

Discordant eye-level grades were distributed to a single experienced trachoma grader (S. K. West) for open adjudication with the primary graders. Concordant eye-level severity data across graders were finalized and aggregated to the person level by selecting the highest severity score of both eyes.

## STATISTICAL ANALYSES

Descriptive statistics were used to examine sample characteristics, prevalence of conjunctival scarring and severity, and other explanatory variables. The prevalence of scarring severity and the corresponding 95% CIs were calculated using simple binomial distribution for specific age groups: 15–19 years, 20–29 years, 30–39 years, 40–49 years, 50–59 years, and 60 years and older. Distributions of scarring prevalence across potential correlates were graphed to inform any further analyses, and differences in proportions were compared by χ^2^ testing when applicable. Investigation of clustering by village and household was conducted using individual random-effects modeling techniques and the resulting interclass correlation coefficients (ICCs) prior to further exploration of predictor variables. Random-effects ordinal logistic regression models were developed to determine associations between demographic, WASH, and district-level trachoma variables with conjunctival scarring with the R ordinal package. To simplify ordinal logistic models, the severity of conjunctival scarring was classified into three levels, ranging from absent, represented by S0, to mild-moderate, represented by S1–S3, to severe, represented by S4. Covariates associated with conjunctival scarring (*P* <0.1) were sequentially added to the multivariable model after adjustment for age. Covariates were retained if found to be statistically significant (*P* <0.05) based on the maximum likelihood estimation. District-level clustering was accounted for with a random-effect term. Potential effect modification of age and sex was evaluated using an interaction term after adjustment for other covariates. Sensitivity analyses were conducted to examine the impact on the final model after excluding individuals with the end stages of trachoma (TT and CO). Data analysis was conducted in R v. 4.2.2.

## RESULTS

Within the 10 study clusters, conjunctival photographs were collected from 729 individuals ages 15 years and older (range per cluster: 44–120 individuals). Seventeen (2.3%) individuals were excluded from subsequent analysis owing to poor photograph quality. The mean age of individuals with graded photographs (*N* = 712) was 38 years, and 63.2% were female. The district-level TF prevalence at the time of this study ranged from 2.0% in Ambassel to 55.3% in Dahanna, and the district-level prevalence of trachomatous scarring among individuals 15 years and older (by field grade) ranged from 2.5% in Ambassel to 11.5% in Dahanna (Supplemental Table 1). Overall, 384 (26.4%) of 1,453 photograph pairs (i.e., eye-level photographs) required adjudication.

A total of 421 (59.1%) of 712 individuals with a scarring grade had some degree of scarring (S1–S4) (Table 2). Most individuals with scarring were classified as S4 (20.4%), in contrast to the prevalence of S3 (11.2%), S2 (8.3%), and S1 (19.2%). Nineteen cases of TT were identified by field grade among participants with a scarring grade, of whom 16 (84.2%) were identified with severe (S4) scarring, which indicates scarring obscuring more than 90% of the upper tarsal conjunctiva.

**Table 2:** Participant characteristics among those with scarring data (*N* = 712), Amhara, Ethiopia, 2017

			Scarring Present			
Characteristics	Overall	Scarring Absent	S1	S2	S3	S4
*N* (%)	712	291 (40.9)	137 (19.2)	59 (8.3)	80 (11.2)	145 (20.4)
Age, Mean ± SD (years)	38 ± 18.3	37.7 ± 16.6	40.8 ± 18.9	42.6 ± 18.2	40.9 ± 19.5	48.0 ± 18.6
Sex No. (%)*						
Male	260	123 (47.3)	64 (24.6)	18 (6.9)	16 (6.2)	39 (15.0)
Female	447	168 (37.6)	72 (16.1)	41 (9.2)	61 (13.6)	105 (23.5)
WASH Characteristics No. (%)						
Lack of Improved Water Source	180	59 (32.8)	29 (16.1)	17 (9.4)	32 (17.8)	43 (23.9)
Presence of Improved Water Source	532	232 (43.6)	108 (20.3)	42 (7.9)	48 (9.0)	102 (19.2)
Latrine Absent	456	172 (37.7)	77 (16.9)	42 (9.2)	61 (13.4)	104 (22.8)
Latrine Present	256	119 (46.5)	60 (23.4)	17 (6.6)	19 (7.4)	41 (16.0)
Distance to Water Under 30 Minutes	342	135 (39.5)	63 (18.4)	24 (7.0)	40 (11.7)	80 (23.4)
Distance to Water 30 Minutes to 1 Hour	175	88 (50.3)	35 (20.0)	13 (7.4)	12 (6.9)	27 (15.4)
Distance to Water Greater than 1 Hour	195	68 (34.9)	39 (20.0)	22 (11.3)	28 (14.4)	38 (19.5)
Trachomatous Trichiasis No. (%)	19	0 (0.0)	0 (0.0)	1 (5.3)	2 (10.5)	16 (84.2)
Corneal Opacity No. (%)	13	1 (7.7)	0 (0.0)	1 (7.7)	0 (0.0)	11 (84.6)

No. = number; WASH = water, sanitation, and hygiene. Rows may not sum to 100 because of rounding.

* The total sample size for the sex category sums to 707 because five individuals were missing data on sex.

**Figure 1.**
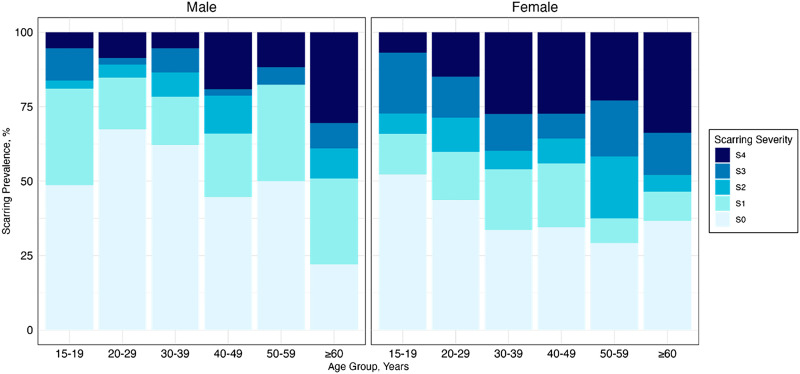
Age-sex–specific distribution of trachomatous scarring severity among adults (ages 15 years and older), Amhara, Ethiopia, 2017.

Scarring at every stage (S1–S4) was observed among even the youngest age group (15–19 years old) (Figure 1). Among older adults (60 years and older), 70.5% (95% CI: 61.9, 78.1) had some stage of scarring. Individuals ages 60 years and older also experienced the greatest burden of severe scarring (S4 prevalence: 32.6%; 95% CI: 24.7, 41.3) compared with the lowest burden among individuals ages 15–19 years (6.2%; 95% CI: 2.0, 13.8). Women were also observed to experience a greater prevalence of scarring at every stage of severity compared with men (Supplemental Tables 2 and 3).

Clustering of scarring prevalence was investigated, with little found at the household level (ICC = 0.004) compared with the village level (ICC = 0.4). Therefore, only village was included as a random effect in modeling. Several individual-, household-, and district-level variables were significantly associated with an increase in category of scarring severity in univariate ordinal logistic regression analyses (Table 3). The odds of increased scarring severity were 0.68 (95% CI: 0.5, 0.91) times lower in the group reporting latrine access than in the group reporting no latrine access. Residing in a household with access to an improved water source was associated with significantly decreased odds of increased scarring severity (odds ratio [OR]: 0.68; 95% CI: 0.50, 0.93) compared with those without improved water access. District-level TF prevalence was also observed to be a significant correlate of increased scarring severity (OR: 1.05; 95% CI: 1.04, 1.06, per 1% increase in prevalence). The interaction term between age and sex was not statistically significant when adjusting for district-level TF prevalence, latrine use, presence of improved water source, and distance to nearest water source.

**Table 3:** Unadjusted random-effects ordinal logistic regression model for trachomatous scarring severity (*N* = 707), Amhara, Ethiopia, 2017

Variable	OR	95% CI
Age, per Year Increase	1.03	1.01, 1.04
Sex		
Male	1.00	–
Female	1.56	1.17, 2.08
Latrine		
Present	0.68	0.51, 0.91
Absent	1.00	–
Improved Water Source		
Yes	0.68	0.50, 0.93
No	1.00	–
Distance to Nearest Water Source		
<30 Minutes	1.00	–
30 Minutes to 1 Hour	0.62	0.44, 0.88
>1 Hour	1.04	0.75, 1.44
District-Level TF Prevalence*	1.05	1.04, 1.06

OR = odds ratio; TF = trachomatous inflammation–follicular. Random-effect term accounted for clustering at the cluster (village) level.

* Per 1% increase in TF.

The final multivariate ordinal logistic regression model demonstrated that sex (OR: 1.77; 95% CI: 1.28, 2.44), age (OR: 1.03; 95% CI: 1.02, 1.04, per year), and district-level TF prevalence (OR: 1.06; 95% CI: 1.01, 1.11, per 1% increase) were factors associated with increased scarring severity after adjustment for each other and village-level clustering (Table 4). Sensitivity analyses indicated that results of the final model did not differ after removing individuals with TT or CO (data not shown).

**Table 4:** Adjusted random-effects ordinal logistic regression model for trachomatous scarring severity (*N* = 707), Amhara, Ethiopia, 2017

Variable	OR	95% CI
Age, per Year Increase	1.03	1.02, 1.04
Sex		
Male	1.00	–
Female	1.77	1.28, 2.44
District-Level TF Prevalence*	1.06	1.01, 1.11

OR = odds ratio; TF = trachomatous inflammation–follicular. Random-effect term accounted for clustering at the cluster (village) level.

* Per 1% increase in TF.

## DISCUSSION

Despite significant uptake of the SAFE strategy in Amhara since 2010 and reductions in clinical signs of TF, conjunctival scarring remains highly prevalent in the region, with most adults having some level of scarring. Much of its older population remains at risk of incident TT. When scarring was present, it was generally severe, with more than one-third comprising the most severe category (S4). This study revealed that increasing age, female sex, and increasing district-level TF prevalence were all independently related to increasing odds of conjunctival scarring severity among adults. Given the magnitude of scarring observed in this study, there will be a need for ongoing TT surveillance, with accompanying TT surgical services, even after the elimination of trachoma as a public health problem in Amhara.

As might be expected in a trachoma-endemic setting, scarring increased with age, with more than 30% of individuals ages 60 years and older observed to have severe (S4) scarring. However, even the youngest individuals in this study, ages 15–19 years, were found to have all levels of scarring, with more than one-quarter of individuals in this age group with scarring of S2 or above. This supports previous findings from the region that in communities where trachoma is highly endemic, scarring begins to form during childhood and adolescence and then continues to worsen as individuals age.^5^^,^^15^ The progression of scarring at younger ages among endemic communities is expected to be a result of both accumulating *C. trachomatis* infection and recurrent inflammation.^4^^,^^15^ Scarring progression and the development of TT have also been observed in the absence of ongoing *C. trachomatis* infection, likely through continual inflammatory processes.^15^^,^^28^ Although districts in Amhara have been receiving annual MDA with antibiotics for up to 8 years, the photographic evidence of increased scarring across the age span, including considerable scarring among those ages 15–19 years, suggests that scarring processes are still driving scarring progression in this region.^24^^,^^25^^,^^29^

A strong association was observed between female sex and scarring severity, which remained after controlling for age and district-level TF prevalence. This reinforces the results of earlier studies that demonstrated a higher likelihood of females developing severe scarring or blindness as a result of trachoma.^12^^,^^13^^,^^30^^–^^32^ It is probable that women are more susceptible to developing scarring because of their societal responsibilities in childcare, which create a greater period of recurrent infection.^33^ However, Wolle et al.^22^ previously reported that the progression of scarring may not be related as much to infection but occurs as a result of biological factors that exacerbate the disease in young and middle-aged women. Given that women have been observed with a higher incidence and severity of autoimmune conditions during their reproductive years and pregnancy and that ocular *C. trachomatis* infection causes an inflammatory immune response leading to conjunctival scarring, there could be a biological similarity within autoimmune diseases and scarring.^34^ Therefore, the association between age and scarring observed in women may be due to biological factors that result in a more severe inflammatory immune response during reproductive years, which then subsides after menopause. Both age and sex were significantly associated with scarring severity after controlling for the other, and it is biologically plausible that these two correlates may act synergistically to increase the risk of developing severe scarring sequelae among women. It has been demonstrated that women are twice as likely to have TT, and these scarring data further demonstrate that even after existing TT cases have been operated on, TT surveillance strategies should prioritize older women.^35^^,^^36^

In this study, the prevalence of TF was used as a measure of current, ongoing *C. trachomatis* transmission; however, it also represented historical exposure. It has been demonstrated in Amhara that current TF is a good marker of TF prevalence levels from earlier in the history of the control program.^24^ Results of the modeling demonstrated that increasing district-level TF prevalence was associated with increasing scarring severity, likely reflecting long-term exposure to transmission. The relationship between *C. trachomatis* infection and scarring incidence varies across communities of varied endemicity. In hyperendemic communities, a significant risk of scarring incidence in adults and children has been associated with constant infection (detection of infection at multiple time points) and severe trachoma.^3^^,^^4^ Scarring incidence is lower in hypo- and formerly hyperendemic settings owing to reduced transmission, but scarring progression can occur even in nonendemic communities.^17^^,^^37^ Although *C. trachomatis* infection may be necessary to start the scarring process, its progression may be inflammatory in nature. In a longitudinal study of the incidence and progression of existing scarring of children in northern Tanzania, Ramadhani et al.^28^ found slim unadjusted associations between infection or TF and scarring progression. In a further study by Burton et al.,^15^ scarring progression was linked to recurrent conjunctival inflammation, potentially influenced by pro-fibrotic factors, suggesting biological susceptibility to prolonged inflammatory episodes in scarring. The results from this study, which demonstrated a correlation between higher district-level TF prevalence and scarring severity, suggest that trachoma control programs may also want to focus surveillance efforts on those districts that were formerly the most highly endemic.

Although several common water and sanitation variables were associated with increased scarring severity in univariate models, they were no longer statistically significant after adjustment for age, sex, and district-level TF prevalence. Currently, few studies have examined the association between water availability and sanitation with scarring. In Tanzania, WASH indicators such as latrine presence and time to water were not statistically significantly associated with incident scarring after controlling for TF.^17^ The WASH characteristics may be too distal in the natural history of trachomatous scarring. Although WASH characteristics describe the setting or environment needed for trachoma transmission, it is the trachoma transmission and associated inflammation that drive scarring processes. It is also likely that the current WASH levels may not be valid reflections of past levels, during the period when the scarring was developing in these participants. Although studies have linked low water and sanitation to higher levels of active trachoma (TF) generally and in Amhara specifically, district-level prevalence of TF was a more important correlate of scarring severity in this model.^20^^,^^29^ Including district-level TF in the model may, in part, have controlled for WASH levels given this link between WASH and trachoma.

The results from this study demonstrate that these districts in Amhara have a considerable population at risk for TT incidence, particularly among women and older age groups. Longitudinal studies have clearly linked scarring to incident TT. In Tanzania, a 7-year TT incidence rate of 1.3% was determined among those with evidence of scarring compared with 0.1% among those without scarring.^7^ In a longitudinal study in the Kilimanjaro region of Tanzania and the Amhara region of Ethiopia, within the Ethiopian cohort, more than 23% of individuals with conjunctival scarring at baseline observed notable progression over a 2-year period.^15^ Such findings imply that scarring sequelae progression is likely to occur independently of other trachoma-related trends in the region and increase the probability of TT development.^15^ The current study also found that most TT cases had severe scarring (S4), reaffirming the link between severe scarring and TT development. A further 31% of the adults in this sample had a scarring grade of S3 or greater, suggesting a population that could be at risk of future TT development. Analytic models have already been developed to understand the relationship between TF and TT and to predict the number of infections needed to develop TT.^38^^,^^39^ Although scarring is currently not used in programmatic decision-making and is no longer assessed among the general population in most surveys, scarring data could help refine models and inform the global trachoma community on the expected burden of incident TT.

This study had some limitations. Photographs for this study came from a sample of 10 clusters surveyed as part of routine trachoma impact surveys; thus, the overall sample size was limited. Clusters included in this sub-study were chosen randomly, and therefore the sample was likely fairly representative.^23^ As cell phone conjunctival photography becomes more common, it will be easier to conduct surveys where larger numbers of individuals can be photographed.^40^ Approximately 2% of photographs were deemed ungradable for scarring in this study, and this may have been due to cooccurrence of follicular and inflammatory signs, which may have prevented full visualization of scarring-related blood vessel obstruction. In addition, a significant proportion (15.6%) of analyzed photographs were classified as grade S1, which is reportedly challenging to grade accurately.^18^ This may have led to nonsystematic misclassification with that score. To address this limitation, multiple graders were used to reach consensus on each photograph. Lastly, although ordinal logistic regression assumes that the relationship between the predictors and the different levels of the response variable is constant, extensive techniques were used to improve interpretation, including variable transformations and subgroup analysis.

This study revealed that nearly 60% of participants had some degree of scarring and that being female, of older age, and living in a district with higher concurrent district-level TF prevalence were independent factors associated with increasing scarring severity. This suggests that a significant portion of the population may be susceptible to scarring progression and TT development, with the potential to exacerbate disparities among subpopulations in Amhara. This further underscores the importance of tailoring public health interventions aimed at eliminating trachoma to meet the specific needs of subpopulations, such as older individuals and particularly older women. Surgical services should target districts with a significant population of aging women, particularly those living in districts with greater TF prevalence levels.

## Supplemental Materials

10.4269/ajtmh.23-0894 Supplemental Materials
