# Supplementary material for: Severity of Trachomatous Scarring among Adults in Trachoma-Endemic Amhara Region of Ethiopia
Source: Am J Trop Med Hyg. 2024 Jun 25;111(3 Suppl):121–6. doi: 10.4269/ajtmh.23-0894 (PMC11376122; doi:10.4269/ajtmh.23-0894)
Supplement: Supplemental Materials [file tpmd230894.SD1.pdf]

Supplemental Figure 1. Surveyed districts as part of the study, Amhara, Ethiopia, 2017.

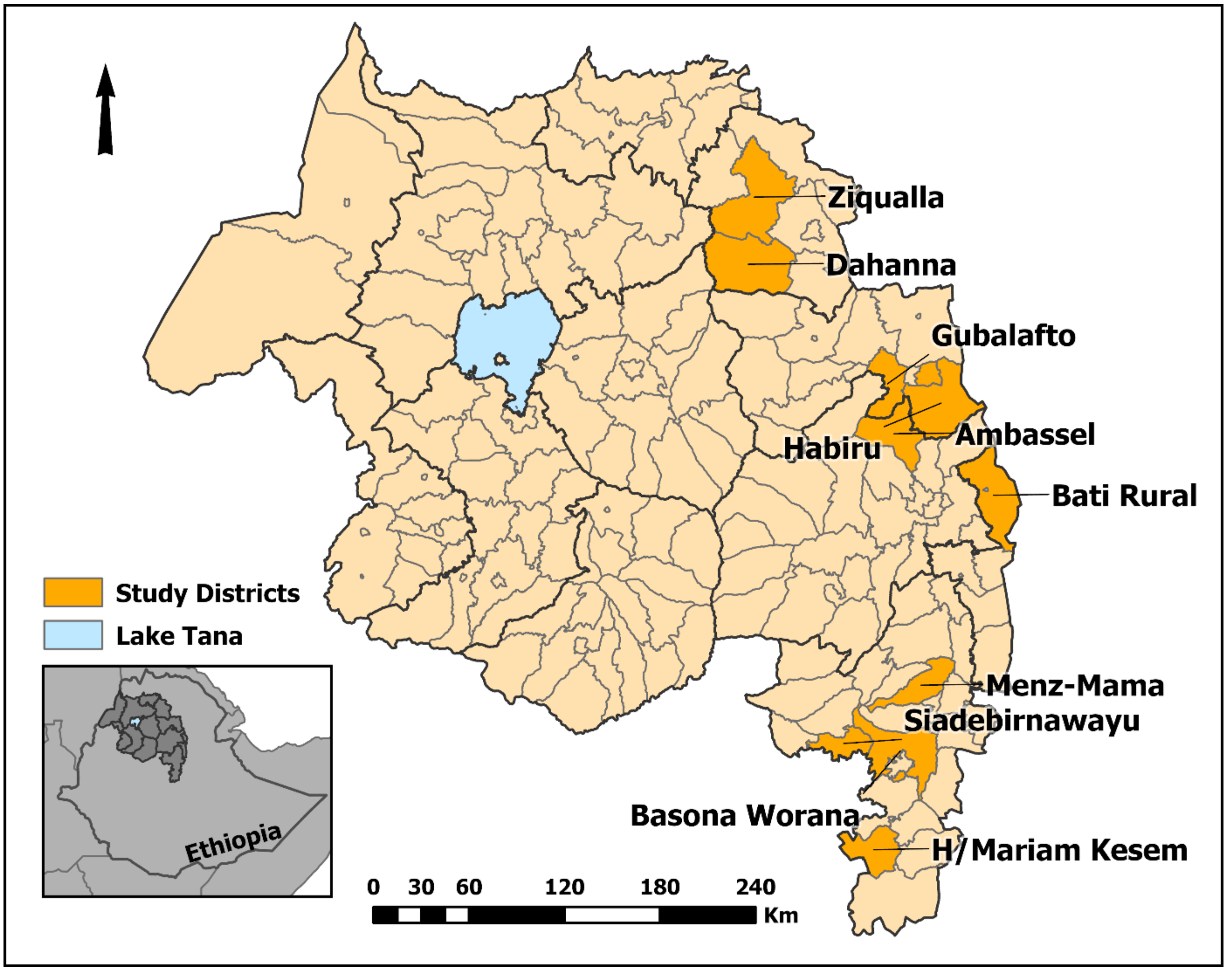

Supplemental Table 1

District-level prevalence of trachomatous inflammation-follicular (TF) among children ages 1 to 9 years and trachomatous scarring (TS) among adults ages 15 and above, Amhara, Ethiopia, 2017

**District Name**	**TF Prevalence %, (95% CI)**	**TS Prevalence %, (95% CI)**
Ambassel	2.0 (0.5, 7.9)	2.5 (1.6, 3.7)
Basona Worana	9.5 (3.8 ,22.1)	4.4 (3.6, 5.3)
Bati Rural	9.9 (4.1, 21.8)	3.4 (2.3, 4.8)
Dahanna	55.3 (49.6, 60.9)	11.5 (9.3, 14.0)
Gubalafto	3.0 (0.8, 9.9)	4.5(3.2, 6.2)
H/Mariam Kesem	15.1 (8.9, 24.5)	7.0 (5.4, 9.0)
Habiru	5.4 (2.3, 12.3)	3.0 (1.9, 4.5)
Menz-Mama	7.2 (3.1, 15.9)	5.9 (5.0, 7.0)
Siadebirnawayu	17.4 (14.8, 20.4)	5.3 (3.8, 7.0)
Ziqualla	16.5 (9.9, 26.4)	5.9 (4.2, 7.9)

CI = confidence interval.

Supplemental Table 2

Age-specific distribution of trachomatous scarring severity among men (ages 15 years and older), Amhara, Ethiopia, 2017

Age category (years)	Overall	Scarring Absent	S1	S2	S3	S4
15-19	37	18 (48.6)	12 (32.4)	1 (2.7)	4 (10.8)	2 (5.4)
20-29	46	31 (67.4)	8 (17.4)	2 (4.3)	1 (2.3)	4 (8.7)
30-39	37	23 (62.2)	6 (16.2)	3 (8.1)	3 (8.1)	2 (5.4)
40-49	47	21 (44.7)	10 (21.3)	6 (12.8)	1 (2.1)	9 (19.1)
50-59	34	17 (50.0)	11 (32.4)	0 (0.0)	2 (5.9)	4 (11.8)
60+	59	13 (22.0)	17 (28.8)	6 (10.2)	5 (8.5)	18 (30.5)

Supplemental Table 3

Age-specific distribution of trachomatous scarring severity among women (ages 15 years and older), Amhara, Ethiopia, 2017

Age category (years)	Overall	Scarring Absent	S1	S2	S3	S4
15-19	44	23 (52.3)	6 (13.6)	3 (6.8)	9 (20.5)	3 (6.8)
20-29	87	38 (43.7)	14 (16.1)	10 (11.5)	12 (13.8)	13 (14.9)
30-39	113	38 (33.6)	23 (20.4)	7 (6.2)	14 (12.4)	31 (27.4)
40-49	84	29 (34.5)	18 (21.4)	7 (8.3)	7 (8.3)	23 (27.4)
50-59	48	14 (29.2)	4 (8.3)	10 (20.8)	9 (18.8)	11 (22.9)
60+	71	26 (36.6)	7 (9.9)	4 (5.6)	10 (14.1)	24 (33.8)
